# From Smelly Buildings to the Scented Past: An Overview of Olfactory Heritage

**DOI:** 10.3389/fpsyg.2021.718287

**Published:** 2022-01-28

**Authors:** Cecilia Bembibre, Matija Strlič

**Affiliations:** UCL Institute for Sustainable Heritage, University College London, London, United Kingdom

**Keywords:** smell, heritage, olfactory, VOC, sensory, intangible, authenticity, system

## Abstract

Olfactory heritage is an aspect of cultural heritage concerning the smells that are meaningful to a community due to their connections with significant places, practices, objects or traditions. Knowledge in this field is produced at the intersection of history, heritage science, chemistry, archaeology, anthropology, art history, sensory science, olfactory museology, sensory geography and other domains. Drawing on perspectives from system dynamics, an approach which focuses on how parts of a system and their relationships result in the collective behaviours of the system, we will outline a series of practices relevant to this field and identify the elements, materials and competences involved, as well as the connections and interactions. While research in olfactory heritage is currently growing, much of the knowledge that could advance our understanding of this field is still being developed within disciplinary boundaries, leading to little integration of the knowledge and methods and limited interdisciplinary interpretation of findings. In the first part, we review the methodologies for identifying, researching and preserving olfactory heritage, highlighting methodological opportunities and challenges from diverse perspectives like smellscape research, odour nuisance management or heritage science. In the second part, we review the presentation and communication of olfactory heritage in museums and other heritage spaces, outlining the value of presenting scents to wide audiences for interpretation and engagement purposes. Finally, we discuss challenges associated with historical scent reconstruction, and discuss future directions for the field, such as the potential of mining large digital collections for olfactory data.

## Introduction

Can we experience a building with our nose? What’s the olfactory equivalent of a painting? Traditionally, these questions would not have made much sense, since our engagement with cultural heritage has relied heavily on visual experiences. A number of recent works, however, explore how olfactory aspects play an important role in the way we understand cultural heritage and therefore make sense of our past and present.

In this article, we will profile the study of the olfactory dimension of heritage, an emerging field of research concerning the smells that are meaningful to a community due to connections with significant places, practices, objects or traditions (Bembibre, 2021). Knowledge in this field is produced at the intersection of history, heritage science, chemistry, archaeology, anthropology, art history, sensory science, olfactory museology, sensory geography and other domains. It relates to tangible, intangible and natural heritage; and it is of interest to a variety of stakeholders, including policy-makers.

Drawing on perspectives from system dynamics, an approach which focuses on how parts of a system and their relationships result in the collective behaviours of the system, we will outline a series of practices relevant to this field and identify the elements, materials and competences involved, as well as the connections, disconnections and interactions. The holistic understanding fostered by the systems theory is especially beneficial to mapping the new and complex landscape of olfactory heritage because it provides a new way of studying phenomena that cross disciplinary boundaries (Forrester, 2007).

## System Dynamics and Olfactory Heritage

**FIGURE 1:**
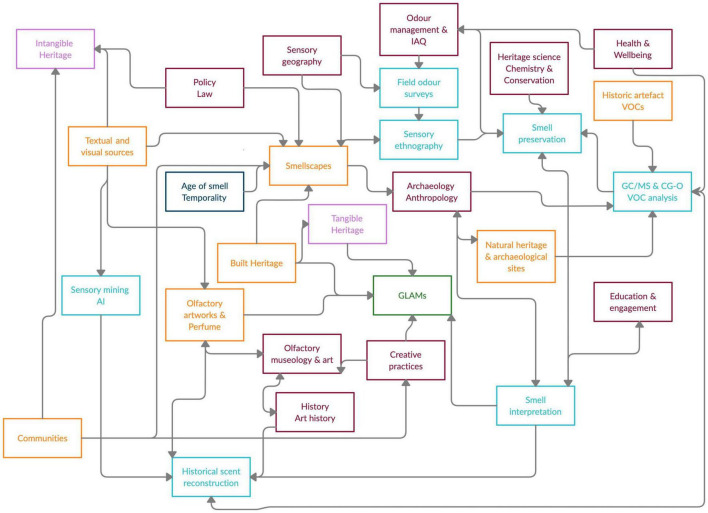
Representation of olfactory heritage as a system, encompassing competences, materials and meanings. The colours indicate dimensions, as follows: maroon: disciplines and knowledge domains; blue: competences and skills; orange: materials, resources and technologies; navy: time; green: environment: purple: heritage approaches. Arrows represent communication and relationship lines.

A systems approach has been applied to study various aspects of cultural heritage, including decision-making concerning historic buildings (Claude et al., 2019) and heritage values and conservation (Fouseki et al., 2020). The study of smells as part of cultural heritage can be related to the “sensory turn” in the humanities in the 1980s and 1990s, which involves “a cultural approach to the study of the senses and a sensory approach to the study of culture” (Howes, 2013). In recent years, scholarly work has contributed to our understanding of the role that odours and our perception of them play in experiencing the world. Olfactory heritage assumes the value of smells as expressions of heritage associated with information, meaning and emotion. Here we focus on (a) the identification and research of the olfactory dimension of historic narratives, material artefacts, practices and geographical places, and (b) the presentation and communication of scents in galleries, libraries, archives and museums (GLAMs). We identify the components of the olfactory heritage system, such as a “material” dimension with resources, artefacts and technologies; associated competencies (skills, know-how and techniques); relevant environments and time; as well as links between these elements (Figure 1). The smells in this system can be textual or visual references, digital or anchored in material documents, as a mix of odorous volatile organic compounds (VOCs) emitted from an historic object, space or a result of a practice. Additionally, they can also be fragrant solutions intended to be smelled, a diverse category that encompasses perfume, historical scent reconstruction, and pieces of olfactory art, among others.

While valuable reflections on olfactory heritage are taking place, much of the knowledge that could advance our understanding of this field is still developed within disciplinary boundaries. For example, the smell of old books and historic libraries, a scent identified as of heritage value (Bembibre and Strlič, 2017), has proved of interest to chemistry and heritage science (Strlič et al., 2009; Cincinelli et al., 2016), architecture and conservation (Shelton and Stuth, 2018), psychology and sensory science (Spence, 2020a), literature and cultural studies (Groes and Francis, 2021), sensory geography (McLean, 2017), public engagement (Wiggan, 2020) and olfactory art (Inoue, 2017). In spite of this varied interest, there is little integration of the knowledge and methods involved in these pieces of research, and limited interdisciplinary interpretation of findings.

Therefore, one of the aims of our approach is to identify those domains of interest for this field and ways in which bridges can be built to deepen the understanding of and develop shared methodologies and practices around olfactory heritage.

## Identifying and Researching Olfactory Heritage

A traditional approach to the protection of cultural heritage considers smells to be sensory qualities of the environment, including significant places, and as such worthy of conservation (ICOMOS, 1988). In this sense, the smells of heritage places can be considered part of their identity. By sniffing we develop a relationship with the environment, engaging in an embodied experience of heritage, evoking memories (Simonnot and Siret, 2014) and leading to shared experiences and a sense of cohesion (Gorman, 2017). Scents also provide a sense of wellbeing and dignity to local communities and tourists, as argued by the selection of 100 “fragrant places” of Japan by popular vote, including parks, bookshops and sake breweries (Ministry for the Environment, 2001).

It has been proposed that smells can also undergo a process of patrimonialisation, becoming part of cultural heritage by being officially presented as identity traits of a place, as it happened with the scent of flowers, woods and livestock representing the Swiss Alpine landscape in an international exhibition (Fraigneau, 2021). Olfactory heritage, and the history and meanings it represents, can also be named by legal means, as in the French “sensory law” protecting the smells of the rural landscape (2021), which goes beyond the fragrant to include malodours (Guy, 2021).

In order to understand the role of smells in people’s perceptions and interpretations of a space, the concept of smellscape, consisting of a distinct set of odours in an environment (Porteous, 1985), is essential. In the last decade, researchers in urban studies and sensory geography (Balez, Bouchard, Fraigneau, Henshaw, McLean, Xiao, among others), have enhanced our knowledge of smellscapes and developed novel methodologies that account for the temporality and subjective nature of odours, recording associations with place, identity and individual experiences and memories. The resulting data, for example odour descriptors collected during smellwalks, is rich and original, and of interest to sensory and heritage science. One of the challenges is, however, to compare descriptions of smellscape obtained via sensory ethnography methods with those collected through odour nuisance analysis (Suffet and Rosenfeld, 2007) or with instrumentally-generated data (e.g., using gas chromatography-olfactometry), as the vocabularies are not standardised. This is why dedicated concepts such as the Porteus (1990, p. 26) smellmark, an olfactory type of landmark, can be extremely helpful. In this line, a smellmark in Istanbul would be the fragrant spice market, recently recorded by Davis and Thys-Şenocak (2017).

While the smells of historic buildings are considered part of their significance, other scents of value can be understood from the perspective of intangible cultural heritage (ICH), which concerns “practices, representations, expressions, knowledge, skills” (UNESCO, 2020). In this context, the scents of Grasse, France, are recognised for their connection with the cultivation of perfume plants, the knowledge and processing of natural raw materials and perfume composition (UNESCO, 2018). Grasse’s inscription was developed with local museums, recording scents’ role in transmitting local knowledge and skills, the involvement of the relevant communities and the need for urgent safeguarding. Learning from the Grasse experience with ICH could prevent meaningful scents from being lost; yet, odours still sit uncomfortably within categories of tangible and intangible heritage, as Boswell (2008) has noted. This is a larger and necessary discussion for those interested in safeguarding olfactory heritage.

Our knowledge of smellscapes and scents of cultural value is informed by research on the sensory worlds of the past. Entering historic sources with an “olfactory gaze,” a term coined by Verbeek C. L. et al. (2020) building on the work of scholars such as David Howes and Constance Classen, is key to understand aspects of olfactory heritage. Examples of the value of this approach are new interpretations of (a) archaeological findings, like the preference for accessibility in spite of experiencing malodours in the design of Roman toilets (Koloski-Ostrow, 2015; p. 39), (b) the symbolism of odours in Egyptian culture, where the smell of fish signified stench but could also imply the danger of an unfamiliar place (Goldsmith, 2019), and (c) the historic role of fragrance, such as the a ritual cleaning of women, in the communities of Zanzibar (Boswell, 2008). In the process of revealing alternative historic narratives, these works often provide us with new perspectives to explore and study olfactory heritage.

Textual and visual sources can offer, as we have shown, valuable clues to capturing and interpreting the smells of the past. Sometimes, these clues are supported by material evidence, such as historic artefacts. Heritage science work on the smell emitted by historic objects has traditionally focussed on potential damage of volatile organic compounds (VOCs), the chemicals responsible for most odours, to collections and visitors. Recent research shows, however, that the presence of certain VOCs in a museum, in concentrations detectable by the human nose, does not pose a threat to collections and can provide valuable information on material change (Strlič et al., 2009; Curran et al., 2018). Laboratory analysis of the smells is also of interest to archaeologists, who can use the data, along textual sources, to inform their interpretations of historic objects and as the basis for smell recreations (Verbeek, 2016, 2019; Newstead and Casimiro, 2020).

A stinking artefact can also be a provocation: after smelling a collar reeking of its owner Rex, a decorated rescue dog, conservators at the Imperial War Museum considered eliminating the stench, but agreed that the olfactory dimension of the object “was authentic” and “emitted Rex’s aura” (Hetherington, 2020). Historic materials smell as they degrade – as the above example shows, there is a tension between the interpretive potential of a “smelly” object and conservation concerns about material condition, VOC impact and risk. Also at play might be the association of all environmental smells with poor air quality, a notion influenced by the historic miasma theory, which identified odours as carriers of diseases. Although discredited by the end of the 19th century, these beliefs maintained a place into popular culture for many decades (Hsu, 2016).

Once the smells of historic objects are identified as valuable, preserving them enables safeguarding their significance. A first step aimed to build bridges between textual sources, analytical and sensory characterisation, resulting in data which can be co-interpreted by different heritage professionals was recently proposed (Bembibre and Strlič, 2017). Further studies could combine this preservation framework with ethnographic methods involving relevant stakeholders, with the aim of revealing and recording the significance of the characterised smells. Identifying narratives related to odours is also essential to building our understanding of their cultural and historic significance, since smells can be “an important medium for understanding the affective capacities of air” Hsu (2016).

## Presenting and Communicating Olfactory Heritage

Many museums, which are traditionally vision-centred spaces, are reluctant to introduce an olfactory art element, exhibiting “anosmic cube” mentality (Drobnick, 2005). Other examples of built heritage face similar challenges: projects to conserve the smell of historic buildings are met with resistance around presenting culturally significant historic scents, such as stale cigarette smoke, in the original space (Otero-Pailos, 2008) and a reproduction of a fragrant, historically relevant component (pot-pourri) is kept cordoned off in an exhibition in a historic house preventing the audience from smelling it (Bembibre Jacobo et al., 2017). In these spaces, smells are viewed with suspicion because they disrupt the climate control and sensory calm to interact with and invade artefacts and visitors’ bodies in uncontrollable ways, even if, as discussed earlier, concentrations are low and pose no obvious health risks. In this respect, Hsu (2020) notes that one aspect of olfactory art is to allude – and challenge – the idea of toxicity and acceptable risk.

In spite of the tensions between conservation and interpretation of historic scents and the visual tradition of the heritage sector, there is a growing number of institutions which work or would like to work with smell [for some examples of exhibitions see Spence (2020b) and Deramond and Pianezza (2020)].

In museums, smells can be a new strategy to respond to visitors’ expectations of immersive experiences that appeal to the audience emotions and senses. Young audiences, in particular, are influenced by ambiance and aesthetics, including temperature, lighting, music and scent (Hyun et al., 2018). In a series of interviews with olfactory museum exhibition designers and visitors, Deramond and Pianezza (2020) noted that the aims of these experiences were to connect artworks, smells and the public, enabling visitors to feel deep attachment to pieces of art and evoke autobiographical memories. As a result, audiences are encouraged to share their experiences of the visit, so smells, and the associated emotions, become a tool to develop and strengthen social bonds.

Odours are also important in heritage interpretation: visitors appreciate the scent of a historic library, imbuing it with symbolic meaning –“inhaling wisdom” – (Bembibre and Strlič, 2017); archives and historic house audiences value the sensory qualities of the documents they access, including the smell (Dillon et al., 2013).

Furthermore, scents are also used to provide alternative ways to engage with collections. In her work at the Rijksmuseum, art historian Verbeek guided an experience of the large painting *The Battle of Waterloo* by Jan Willem Pieneman (1984)^1^. To relate the size of the canvas to her blind audience, she slowly walked its length with audible footsteps. Later, visitors smelled an interpretation of the odour of the battle developed with IFF perfumer, Birgit Sijbrands. A person noted that “the scents really contributed to the stories surrounding the objects, not the artefacts themselves. Being 100% blind I do not feel any connection to – for example – paintings. The scents really made me empathise with people from different eras though” (Verbeek C., 2020).

Presenting and communicating smells with historic and cultural value to wide audiences is a challenge due to the lack of standardised approaches or best practices. Historic perfumes are either reconstructed from the original formula, if such exists (and thousands do, in archives such as the Osmothèque) or, if the formula were inaccessible, recreated from references in texts or images, or from data obtained by analysing historical odour sources such artefacts or materials. In these cases, a growing, and, in many instances, exploratory, practice is being developed at the intersection of academia and industry, which has resulted in many past smells being recreated for the contemporary nose (see Verbeek, 2016, 2019; Barwich and Rodriguez, 2020; Bembibre, 2021; Littman et al., 2021). Discussions around the authenticity of such recreations evidence the tensions between traditional study of history and historic artefacts and approaches which, in a similar way to battle re-enactions, foster affective and sensory experiences, making a distant heritage seem “real and tangible” (Mullins, 2013). Currently, these considerations, along with those around authorship and audience manipulation, tend to take place in the realm of curatorship (Drobnick, 2017). These issues could also be usefully explored in relation to the development of smell (re)creations and the public experiencing them. Additionally, the related issue of presenting original historic objects for smelling, or purposedly scented proxies, in a museum context, is one that requires similar investigation.

Fruitful connections could be made by encouraging GLAMs and archaeological sites to document the experiences of visitors in relation to olfactory exhibits, since most of the available data for the use of ambient scent on people’s responses to art has been obtained in laboratory studies and may not be directly generalisable to the museum floor (Spence, 2020a). Furthermore, new methods for the development of olfactory narratives would facilitate a “nose-first” approach and new interpretations of existing collections and spaces. In addition, the development, jointly with conservation organisations and industry bodies, of best practices to present and communicate smells, such as indoor air quality (IAQ) standards, smell diffusion methods and public liability disclaimers, would encourage reluctant institutions to engage in sensory work and get a clear understanding of the risks and benefits involved.

Finally, the valuable role of art and creative practices in the development and communication of olfactory heritage is worth noting. For example, the pioneering work of olfactory artists such as Sissel Tolaas and Peter de Cupere, who interrogate the way we experience and discuss scent today; the “Booksniffers Club,” a series of workshops funded by Arts Council England where participants smell books in their own collections and share the memories they evoke, leading to conversations about history, culture and identity (Wiggan, 2020) or the “Kitchen Cabinet” sessions by Dutch cultural centre Mediamatic, providing olfactory experiences in which participants learn how the contents of their spice drawers hold meaningful connections to the history of art (Mediamatic, 2021) and the series of lectures and interactive sessions educating about global olfactory practices carried out by the Los Angeles-based The Institute for Art and Olfaction (2020).

## Future Directions

Access to digital collections of historic texts and images using artificial intelligence techniques has recently enabled the discovery of olfactory references within large data sets, i.e., sensory mining. Tracing historic references to odours in collections, and working with sensory historians to identify and build up the narratives that relate them to the present, is one of the goals of the interdisciplinary project Odeuropa, which focuses on capturing olfactory heritage across multiple European regions and languages from the 17th to the 20th century and then present them in museums (Odeuropa, 2021). In this project, historians, anthropologists and scientists work together, a collaborative mode that could lead to further methodological development and new approaches to co-interpretation. Polish-Slovene project Odotheka also explores semlls in heritage, developing an archive of historic smells from the collections of the National Museum of Krakow, and the National Museum of Slovenia (Odotheka, 2021).

In this article, we’ve identified some additional directions for future work, which could develop further connections and interactions between elements in the olfactory heritage system, evolving and linking practices. With regards to smell preservation, the integration of analytical work with ethnographic approaches, anchored in written and visual sources when available, would strengthen the process of studying smells in buildings and artefacts, while encouraging discussions around complex historical and social narratives. In order to effectively work with smell in GLAMs for interpretive, accessibility or educational purposes, collecting real world data would validate laboratory studies and deepen understanding. Widening the debate to all stakeholders in this system would enable wider engagement with the ethical dimension of the work. Finally, working with industry and heritage bodies would be beneficial to the development of best practice and safety guidelines around communicating olfactory heritage.

## Author Contributions

The authors conceived the present review as a reflection of historic discussions between them based on their work in the field. Both authors discussed the literature, contributed to the final manuscript, and approved it for publication.

## Conflict of Interest

The authors declare that the research was conducted in the absence of any commercial or financial relationships that could be construed as a potential conflict of interest.

## Publisher’s Note

All claims expressed in this article are solely those of the authors and do not necessarily represent those of their affiliated organizations, or those of the publisher, the editors and the reviewers. Any product that may be evaluated in this article, or claim that may be made by its manufacturer, is not guaranteed or endorsed by the publisher.

## Footnotes

1
https://www.rijksmuseum.nl/en/collection/SK-A-1115
